# Supplementary material for: Surveillance and Characterization of Vancomycin-Resistant and Vancomycin-Variable Enterococci in a Hospital Setting
Source: Antibiotics (Basel). 2025 Aug 4;14(8):795. doi: 10.3390/antibiotics14080795 (PMC12383138; doi:10.3390/antibiotics14080795)
Supplement: Supplementary file 1 [file antibiotics-14-00795-s001.zip › Supplementary Files/Figure S2-antibiotics-3720173.pdf]

Figure S2. Multilocus (ML) phylogenetic tree of the isolates included in our study[1].

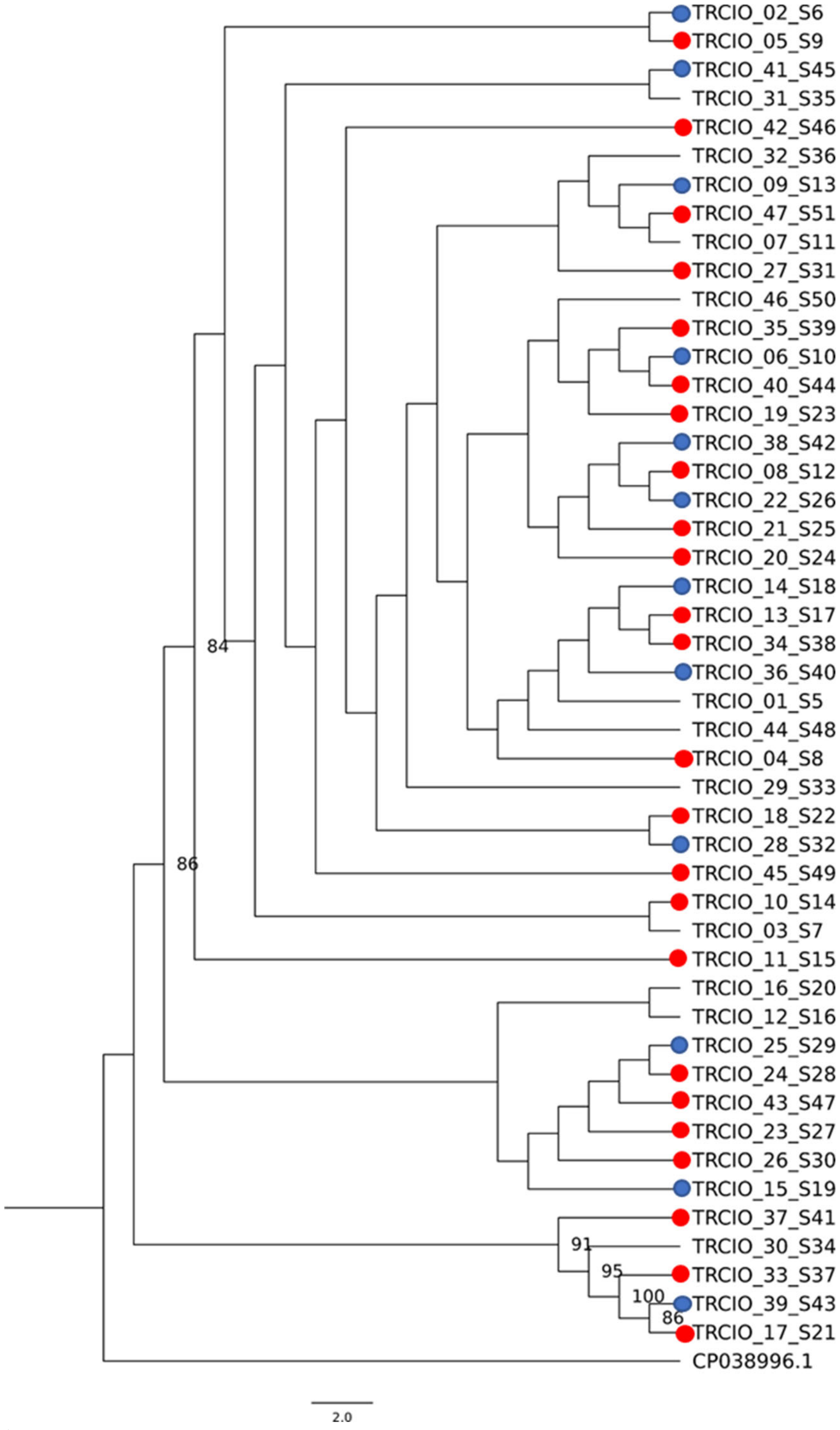

[1]Blue circles indicate colonization prior the admission in our Institute; red circles indicate colonization after 48 hours in our Institution.
