# Supplementary material for: Surveillance and Characterization of Vancomycin-Resistant and Vancomycin-Variable Enterococci in a Hospital Setting
Source: Antibiotics (Basel). 2025 Aug 4;14(8):795. doi: 10.3390/antibiotics14080795 (PMC12383138; doi:10.3390/antibiotics14080795)
Supplement: Supplementary file 1 [file antibiotics-14-00795-s001.zip › Supplementary Files/Figure S3-antibiotics-3720173.pdf]

Figure S3. Minimum Spanning Tree (MST) of VRE and VVE clinical isolates[1].

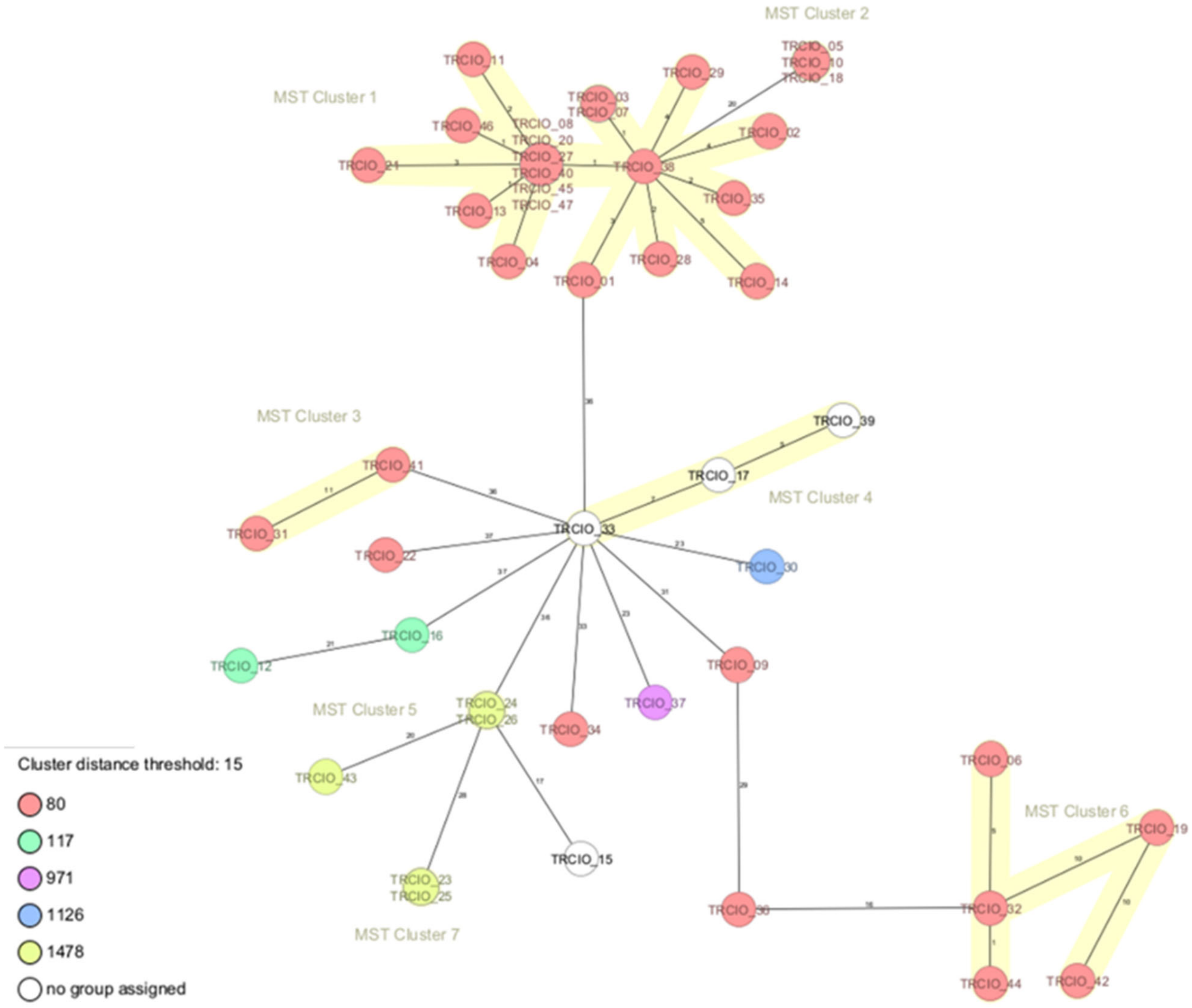

[1]Distance based on columns from *E. faecium* MLST (7), *E. faecium* cgMLST (1423), *E. faecium* Accessory (1124).

MST Cluster distance threshold: 15

Nodes coloured by column: ST

E. faecium cgMLST Complex Type/Clustering Distance: 20
