# Supplementary material for: Surveillance and Characterization of Vancomycin-Resistant and Vancomycin-Variable Enterococci in a Hospital Setting
Source: Antibiotics (Basel). 2025 Aug 4;14(8):795. doi: 10.3390/antibiotics14080795 (PMC12383138; doi:10.3390/antibiotics14080795)

Figure S4. Workflow diagram for the identification and confirmation of VRE and VVE from rectal swabs using MA, selective media, and WGS.

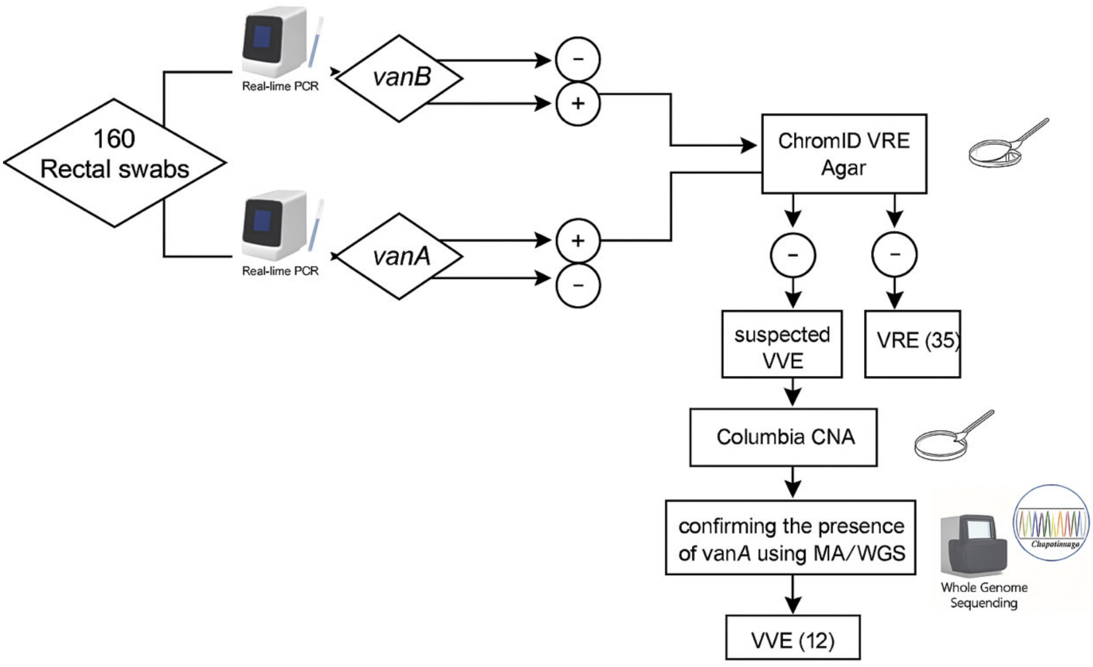

Supplement: Supplementary file 1 [file antibiotics-14-00795-s001.zip › Supplementary Files/Figure S4-antibiotics-3720173.pdf]
