# Supplementary material for: Surveillance and Characterization of Vancomycin-Resistant and Vancomycin-Variable Enterococci in a Hospital Setting
Source: Antibiotics (Basel). 2025 Aug 4;14(8):795. doi: 10.3390/antibiotics14080795 (PMC12383138; doi:10.3390/antibiotics14080795)
Supplement: Supplementary file 1 [file antibiotics-14-00795-s001.zip › Supplementary Files/Table S1-antibiotics-3720173.pdf]

Table S1. The complete list of the *E. faecium* strains, together with their total number of reads, QUAST assembly statistics, genome BUSCO completeness and annotation

Assembly	Organism	N. of paired end reads	N. of contigs	Largest contig	Total lenght	Bases	GC (%)	N50	N90	L50	L90	BUSCO completeness	N. of CDS	r RNA	t RNA	tm RNA	CRISPR
TRCIO_01_S5	*Enterococcus faecium*	2757408	270	135240	2909195	2925710	38.17.00	35832	8070	24	91	98.4%	2812	4	56	1	
TRCIO_02_S6	*Enterococcus faecium*	3502590	255	135241	2875285	2889668	38.17.00	38914	7305	23	89	98.4%	2768	3	56	1	
TRCIO_03_S7	*Enterococcus faecium*	2980806	258	135241	2976210	2991574	38.05.00	42839	7756	23	86	98.4%	2909	4	57	1	
TRCIO_04_S8	*Enterococcus faecium*	3574188	282	129079	3006939	3024459	38.02.00	41903	7633	23	93	98.4%	2955	3	57	1	
TRCIO_05_S9	*Enterococcus faecium*	3548304	260	135241	2993424	3008607	38.02.00	41903	8108	23	91	98.4%	2926	4	56	1	
TRCIO_06_S10	*Enterococcus faecium*	2913090	237	160405	2899831	2913789	38.07.00	39604	9958	19	75	98.4%	2780	3	55	1	
TRCIO_07_S11	*Enterococcus faecium*	1978990	263	135240	2974788	2990259	38.04.00	41903	8264	23	85	98.4%	2911	4	57	1	
TRCIO_08_S12	*Enterococcus faecium*	2790454	256	129079	2914772	2928303	38.16.00	42841	8072	22	88	98.4%	2829	3	57	1	
TRCIO_09_S13	*Enterococcus faecium*	3107784	239	160402	2854949	2870506	38.14.00	47793	8132	18	74	98.4%	2711	3	55	1	
TRCIO_10_S14	*Enterococcus faecium*	3148566	243	135241	2890230	2902648	38.16.00	38914	8108	23	87	98.4%	2778	4	56	1	
TRCIO_11_S15	*Enterococcus faecium*	2556444	238	129079	2873803	2886698	38.19.00	45753	7885	21	82	98.4%	2783	3	57	1	
TRCIO_12_S16	*Enterococcus faecium*	2720286	236	129372	2918134	2935766	38.09.00	47474	9446	18	71	98.4%	2803	4	57	1	
TRCIO_13_S17	*Enterococcus faecium*	2969660	248	129079	2916399	2929004	38.16.00	42839	8108	22	85	98.4%	2830	3	57	1	
TRCIO_14_S18	*Enterococcus faecium*	2918422	277	129079	2848259	2866743	38.19.00	36772	7762	24	90	98.4%	2746	3	56	1	
TRCIO_15_S19	*Enterococcus faecium*	2762746	248	145266	2903568	2919888	38.15.00	47475	9054	16	71	98.4%	2774	3	58	1	
TRCIO_16_S20	*Enterococcus faecium*	2961166	260	129133	2992509	3011501	38.05.00	53143	9288	19	70	98.4%	2883	4	60	1	

TRCIO_17_S21	*Enterococcus faecalis*	2964938	59	696145	2941098	2945193	37.38.00	165890	49815	5	18	98.4%	2797	3	47	1
TRCIO_18_S22	*Enterococcus faecium*	2452280	250	135240	2887963	2900820	38.16.00	38914	7605	23	89	98.4%	2778	3	56	1
TRCIO_19_S23	*Enterococcus faecium*	2933300	271	132514	2868309	2890423	38.15.00	46166	8256	19	75	98.4%	2725	3	55	1
TRCIO_20_S24	*Enterococcus faecium*	2647790	265	129079	2903230	2920806	38.17.00	36712	8132	23	87	98.4%	2815	3	57	1
TRCIO_21_S25	*Enterococcus faecium*	2838258	258	95440	2914508	2928778	38.16.00	41903	7762	24	90	98.4%	2828	3	57	1
TRCIO_22_S26	*Enterococcus faecium*	2739426	270	102121	2895898	2912737	38.02.00	33707	7605	27	88	98.4%	2797	3	58	1
TRCIO_23_S27	*Enterococcus faecium*	2783856	244	129133	2874698	2890701	38.18.00	42431	9298	19	69	98.4%	2744	4	58	1
TRCIO_24_S28	*Enterococcus faecium*	3724304	248	129133	2937349	2950721	38.11.00	42571	9054	20	76	98.4%	2811	3	58	1
TRCIO_25_S29	*Enterococcus faecium*	3186830	232	129372	2876248	2892099	38.18.00	42964	11060	18	66	98.4%	2751	3	58	1
TRCIO_26_S30	*Enterococcus faecium*	3023140	253	129133	2937746	2951201	38.11.00	42571	7762	20	79	98.4%	2814	3	58	1
TRCIO_27_S31	*Enterococcus faecium*	3005624	259	129079	2996368	3010886	38.04.00	42839	7762	23	90	98.4%	2825	3	57	1
TRCIO_28_S32	*Enterococcus faecium*	3016796	261	135263	2894170	2908451	38.17.00	42839	8070	22	86	98.4%	2794	3	57	1
TRCIO_29_S33	*Enterococcus faecium*	2722670	341	135244	2901837	2930697	38.14.00	36758	7770	23	91	98.4%	2825	3	56	1
TRCIO_30_S34	*Enterococcus faecium*	2806846	133	197215	2582139	2592182	38.38.00	77741	17667	11	35	98.4%	2488	3	58	1
TRCIO_31_S35	*Enterococcus faecium*	2706088	262	134500	3006679	3025825	38.04.00	50490	8497	19	75	98.4%	2897	4	59	1
TRCIO_32_S36	*Enterococcus faecium*	2697270	216	160405	2874212	2887068	38.13.00	47473	8913	19	71	98.4%	2759	3	57	1
TRCIO_33_S37	*Enterococcus faecalis*	2865890	207	682105	3279214	3304127	37.05.00	161704	25750	6	25	98.4%	3263	4	54	1
TRCIO_34_S38	*Enterococcus faecium*	3344580	267	145493	2891366	2911916	38.05.00	47713	8232	20	74	98.4%	2736	3	54	1
TRCIO_35_S39	*Enterococcus faecium*	3541718	246	135244	2885326	2899306	38.18.00	42839	8676	22	81	98.4%	2801	3	57	1
TRCIO_36_S40	*Enterococcus*	4166556	230	134605	2780504	2795720	38.25.00	45999	8234	18	75	98.4%	2634	3	55	1

	faecium																
TRCIO_37_S41	*Enterococcus faecium*	3788536	193	137242	2705607	2719524	38.31.00	46726	10877	17	61	98.4%	2620	3	59	1	
TRCIO_38_S42	*Enterococcus faecium*	3855486	253	135241	2950836	2962897	38.05.00	38914	8070	23	89	98.4%	2864	3	56	1	
TRCIO_39_S43	*Enterococcus faecalis*	3634922	65	423436	3012205	3016900	37.26.00	205480	66639	5	15	98.4%	2900	3	53	1	1
TRCIO_40_S44	*Enterococcus faecium*	3680884	261	129331	2911470	2927315	38.16.00	42839	7770	22	89	98.4%	2824	3	57	1	
TRCIO_41_S45	*Enterococcus faecium*	3394292	270	134493	2994244	3015467	38.04.00	52453	8389	19	78	98.4%	2889	3	59	1	
TRCIO_42_S46	*Enterococcus faecium*	2627560	311	160405	2971933	2997820	38.04.00	47475	7770	18	76	98.4%	2848	3	57	1	
TRCIO_43_S47	*Enterococcus faecium*	3517118	228	129373	2887132	2901597	38.17.00	47478	9546	16	65	98.4%	2765	3	58	1	
TRCIO_44_S48	*Enterococcus faecium*	3667584	222	160405	2873936	2887217	38.13.00	46222	8913	19	73	98.4%	2760	3	57	1	
TRCIO_45_S49	*Enterococcus faecium*	3580138	259	129332	3017467	3031230	38.01.00	42839	7770	23	90	98.4%	2956	3	57	1	
TRCIO_46_S50	*Enterococcus faecium*	3293654	254	129079	2914013	2927908	38.17.00	42839	8108	22	87	98.4%	2827	3	57	1	
TRCIO_47_S51	*Enterococcus faecium*	3376010	264	129079	2912498	2927942	38.16.00	38704	7770	23	88	98.4%	2817	3	57	1	
