# Supplementary material for: Surveillance and Characterization of Vancomycin-Resistant and Vancomycin-Variable Enterococci in a Hospital Setting
Source: Antibiotics (Basel). 2025 Aug 4;14(8):795. doi: 10.3390/antibiotics14080795 (PMC12383138; doi:10.3390/antibiotics14080795)
Supplement: Supplementary file 1 [file antibiotics-14-00795-s001.zip › Supplementary Files/Table S2-antibiotics-3720173.pdf]

Table S2. Molecular characterization of the Vancomycin-Resistant enterococci (VRE) isolates.

ID VRE Isolates	Glycopeptide Resistance	Additional resistance genes	Virulence determinants	Plasmids
TRCIO_01	*van*HAX	*aac(6')-Ii, ant(6)-Ia, aph(3')-III, aac(6')-aph(2''), msr(C), erm(B), tet(M), tet(L), dfrG*	*IS16, acm, bepA, ccpA, empA, empB, empC, fms11, fms13, fms14, fms15, fms16, fms17, fms19, fms21, fnm, gls20, gls33, glsB, glsB1, hyl, orf1481, ptsD, sagA, scm, sgrA*	RepUS43, RepUS12, RepUS15, Rep17, Rep11a
TRCIO_02	*van*HAX	*aac(6')-Ii, ant(6)-Ia, aph(3')-III, aac(6')-aph(2''), msr(C), erm(B), tet(M), tet(L), dfrG*	*IS16, acm, bepA, ccpA, empA, empB, empC, fms11, fms13, fms14, fms15, fms16, fms17, fms19, fms21, fnm, gls20, gls33, glsB, glsB1, hyl, orf1481, ptsD, sagA, scm, sgrA*	RepUS43, RepUS12, RepUS15 Rep17, Rep11a
TRCIO_04	*van*H, *van*X	*aac(6')-Ii, ant(6)-Ia, aph(3')-III, aac(6')-aph(2''), msr(C), erm(B), tet(M), tet(L), dfrG*	*IS16, acm, bepA, ccpA, empA, empB, empC, fms11, fms13, fms14, fms15, fms16, fms17, fms19, fms21, fnm, gls20, gls33, glsB, glsB1, hyl, orf1481, ptsD, sagA, sgrA*	RepUS43, RepUS12, RepUS15 Rep17, Rep11a
TRCIO_05	*van*HAX	*aac(6')-Ii, ant(6)-Ia, aph(3')-III, aac(6')-aph(2''), msr(C), erm(B), tet(M), tet(L), dfrG*	*IS16, acm, bepA, ccpA, empA, empB, empC, fms11, fms13, fms14, fms15, fms16, fms17, fms19, fms21, fnm, gls20, gls33, glsB, glsB1, hyl, orf1481, ptsD, sagA, scm, sgrA*	RepUS43, RepUS12, RepUS15 Rep17, Rep11a
TRCIO_06	*van*HAX	*aac(6')-Ii, ant(6)-Ia, aph(3')-III, aac(6')-aph(2''), msr(C), erm(B), erm(T), tet(M), tet(L)*	*IS16, acm, bepA, ccpA, empA, empB, empC, fms11, fms13, fms14, fms15, fms16, fms17, fms19, fms21, fnm, gls20, gls33, glsB, glsB1, hyl, orf1481, ptsD, sagA, scm, sgrA*	RepUS43, RepUS12, RepUS15 Rep17, Rep11a, Rep18b
TRCIO_08	*van*HAX	*aac(6')-Ii, ant(6)-Ia, aph(3')-III, aac(6')-aph(2''), msr(C), erm(B), tet(M), tet(L), dfrG*	*IS16, acm, bepA, ccpA, empA, empB, empC, fms11, fms13, fms14, fms15, fms16, fms17, fms19, fms21, fnm, gls20, gls33, glsB, glsB1, hyl, orf1481, ptsD, sagA, scm, sgrA*	RepUS43, RepUS12, RepUS15 Rep17, Rep11a
TRCIO_09	*van*HAX	*aac(6')-Ii, ant(6)-Ia, aph(3')-III, aac(6')-aph(2''), msr(C), erm(B), erm(T), tet(M), tet(L)*	*IS16, acm, bepA, ccpA, empA, empB, empC, fms11, fms13, fms14, fms15, fms16, fms17, fms19, fms21, fnm, gls20, gls33, glsB, glsB1, hyl, orf1481, ptsD, sagA, scm, sgrA*	RepUS43, RepUS12, RepUS15, Rep17, Rep11a
TRCIO_10	*van*HAX	*aac(6')-Ii, ant(6)-Ia, aph(3')-III, aac(6')-aph(2''), msr(C), erm(B), tet(M), tet(L), dfrG*	*IS16, acm, bepA, ccpA, empA, empB, empC, fms11, fms13, fms14, fms15, fms16, fms17, fms19, fms21, fnm, gls20, gls33, glsB, glsB1, hyl, orf1481, ptsD, sagA, scm, sgrA*	RepUS43, RepUS12, RepUS15 Rep17, Rep11a, Rep14a
TRCIO_11	*van*HAX	*aac(6')-Ii, ant(6)-Ia, aph(3')-III, aac(6')-aph(2''), msr(C), erm(B), tet(M), tet(L), dfrG*	*IS16, acm, bepA, ccpA, empA, empB, empC, fms11, fms13, fms14, fms15, fms16, fms17, fms19, fms21, fnm, gls20, gls33, glsB, glsB1, hyl, orf1481, ptsD, sagA, sgrA*	RepUS43, RepUS12, RepUS15 Rep17, Rep11a
TRCIO_12	*van*HAX	*aac(6')-Ii, ant(6)-Ia, aph(3')-III, aac(6')-aph(2''), msr(C), erm(B), dfrG*	*IS16, acm, bepA, ccpA, ecbA, empA, empB, empC, fms11, fms13, fms14, fms15, fms16, fms17, fms19, fms21, fnm, gls20, gls33, glsB, glsB1, hyl, orf1481, ptsD, sagA, sgrA*	Rep14a, Rep14b, Rep2 Rep17, RepUS15, Rep18a
TRCIO_13	*van*HAX	*aac(6')-Ii, ant(6)-Ia, aph(3')-III, aac(6')-aph(2''), msr(C), erm(B), tet(M), tet(L), dfrG*	*IS16, acm, bepA, ccpA, empA, empB, empC, fms11, fms13, fms14, fms15, fms16, fms17, fms19, fms21, fnm, gls20, gls33, glsB, glsB1, hyl, orf1481, ptsD, sagA, sgrA*	RepUS43, RepUS12, RepUS15 Rep17, Rep11a
TRCIO_14	*van*HAX	*aac(6')-Ii, ant(6)-Ia, aph(3')-III, aac(6')-aph(2''), msr(C), erm(B), tet(M), tet(L), dfrG*	*IS16, acm, bepA, ccpA, empA, empB, empC, fms11, fms13, fms14, fms15, fms16, fms17, fms19, fms21, fnm, gls20, gls33, glsB, glsB1, hyl, orf1481, ptsD, sagA, scm, sgrA*	RepUS43, RepUS12, RepUS15 Rep17, Rep11a
TRCIO_15	*van*HAX	*aac(6')-Ii, ant(6)-Ia, aph(3')-III, aac(6')-aph(2''), msr(C), erm(B), dfrG*	*IS16, acm, bepA, ccpA, ecbA, empA, empB, empC, fms11, fms13, fms14, fms15, fms16, fms17, fms19, fms21, fnm, gls20, gls33, glsB, glsB1, hyl, orf1481, ptsD, sagA, scm, sgrA*	Rep17, RepUS15

TRCIO_18	*van*HAX	*aac(6')-Ii, ant(6)-Ia, aph(3')-III, aac(6')-aph(2''), msr(C), erm(B), tet(M), tet(L), dfrG*	*IS16, acm, bepA, ccpA, empA, empB, empC, fms11, fms13, fms14, fms15, fms16, fms17, fms19, fms21, fnm, gls20, gls33, glsB, glsB1, hyl, orf1481, ptsD, sagA, scm, sgrA*	RepUS43, RepUS12, RepUS15 Rep17, Rep11a
TRCIO_19	*van*HAX	*aac(6')-Ii, ant(6)-Ia, aph(3')-III, aac(6')-aph(2''), msr(C), erm(B), tet(M), tet(L)*	*IS16, acm, bepA, ccpA, empA, empB, empC, fms11, fms13, fms14, fms15, fms16, fms17, fms19, fms21, fnm, gls20, gls33, glsB, glsB1, hyl, orf1481, ptsD, sagA, scm, sgrA*	RepUS43, RepUS12, RepUS15 Rep17, Rep11a, Rep18a, Rep18b
TRCIO_20	*van*HAX	*aac(6')-Ii, ant(6)-Ia, aph(3')-III, aac(6')-aph(2''), msr(C), erm(B), tet(M), tet(L), dfrG*	*IS16, acm, bepA, ccpA, empA, empB, empC, fms11, fms13, fms14, fms15, fms16, fms17, fms19, fms21, fnm, gls20, gls33, glsB, glsB1, hyl, orf1481, ptsD, sagA, sgrA*	RepUS43, RepUS12, RepUS15 Rep17, Rep11a
TRCIO_21	*van*HAX	*aac(6')-Ii, ant(6)-Ia, aph(3')-III, aac(6')-aph(2''), msr(C), erm(B), tet(M), tet(L), dfrG*	*IS16, acm, bepA, ccpA, empA, empB, empC, fms11, fms13, fms14, fms15, fms16, fms17, fms19, fms21, fnm, gls20, gls33, glsB, glsB1, hyl, orf1481, ptsD, sagA, sgrA*	RepUS43, RepUS12, RepUS15 Rep17, Rep11a
TRCIO_22	*van*HAX	*aac(6')-Ii, ant(6)-Ia, aph(3')-III, aac(6')-aph(2''), msr(C), erm(B), cat, tet(M), tet(L), dfrG*	*IS16, acm, bepA, ccpA, empA, empB, empC, fms11, fms13, fms14, fms15, fms16, fms17, fms19, fms21, fnm, gls20, gls33, glsB, glsB1, orf1481, ptsD, sagA, sgrA*	Rep11a, Rep2, Rep17, RepUS15
TRCIO_23	*van*HAX	*aac(6')-Ii, ant(6)-Ia, aph(3')-III, aac(6')-aph(2''), msr(C), erm(B), dfrG*	*IS16, acm, bepA, ccpA, ecbA, empA, empB, empC, fms11, fms13, fms15, fms16, fms17, fms19, fms21, fnm, gls20, gls33, glsB, glsB1, hyl, orf1481, ptsD, sgrA*	Rep17, RepUS15, Rep29
TRCIO_24	*van*HAX	*aac(6')-Ii, ant(6)-Ia, aph(3')-III, aac(6')-aph(2''), msr(C), erm(B), dfrG*	*IS16, acm, bepA, ccpA, ecbA, empA, empB, empC, fms11, fms13, fms14, fms15, fms16, fms17, fms19, fms21, fnm, gls20, gls33, glsB, glsB1, hyl, orf1481, ptsD, sagA, sgrA*	Rep17, RepUS15, Rep11a, Rep18b, Rep29
TRCIO_25	*van*HAX	*aac(6')-Ii, ant(6)-Ia, aph(3')-III, aac(6')-aph(2''), msr(C), erm(B), dfrG*	*IS16, acm, bepA, ccpA, ecbA, empA, empB, empC, fms11, fms13, fms14, fms15, fms16, fms17, fms19, fms21, fnm, gls20, gls33, glsB, glsB1, hyl, orf1481, ptsD, sgrA*	Rep17, RepUS15, Rep29
TRCIO_26	*van*HAX	*aac(6')-Ii, ant(6)-Ia, aph(3')-III, aac(6')-aph(2''), msr(C), erm(B), dfrG*	*IS16, acm, bepA, ccpA,ecbA, empA, empB, empC, fms11, fms13, fms14, fms15, fms16, fms17, fms19, fms21, fnm, gls20, gls33, glsB, glsB1, hyl, orf1481, ptsD, sagA, sgrA*	Rep11a, Rep18b, Rep29, Rep17, RepUS15
TRCIO_27	*van*HAX	*aac(6')-Ii, ant(6)-Ia, aph(3')-III, aac(6')-aph(2''), msr(C), erm(B), tet(M), tet(L), dfrG*	*IS16, acm, bepA, ccpA, empA, empB, empC, fms11, fms13, fms14, fms15, fms16, fms17, fms19, fms21, fnm, gls20, gls33, glsB, glsB1, hyl, orf1481, ptsD, sagA, scm, sgrA*	RepUS43, RepUS12, RepUS15, Rep17, Rep11a
TRCIO_28	*van*HAX	*aac(6')-Ii, ant(6)-Ia, aph(3')-III, aac(6')-aph(2''), msr(C), erm(B), tet(M), tet(L), dfrG*	*IS16, acm, bepA, ccpA, empA, empB, empC, fms11, fms13, fms14, fms15, fms16, fms17, fms19, fms21, fnm, gls20, gls33, glsB, glsB1, hyl, orf1481, ptsD, sagA, sgrA*	RepUS43, RepUS12, RepUS15, Rep17, Rep11a
TRCIO_31	*van*HAX	*aac(6')-Ii, ant(6)-Ia, aph(3')-III, aac(6')-aph(2''), msr(C), erm(B), tet(M)*	*IS16, acm, bepA, ccpA, ecbA, empA, empB, empC, fms11, fms13, fms14, fms15, fms16, fms17, fms19, fms21, fnm, gls20, gls33, glsB, glsB1, hyl, orf1481, ptsD, sagA, sgrA*	Rep11a, Rep17, Rep15
TRCIO_34	*van*HAX	*aac(6')-Ii, ant(6)-Ia, aph(3')-III, aac(6')-aph(2''), msr(C), erm(B)*	*IS16, acm, bepA, ccpA, empA, empB, empC, fms11, fms13, fms14, fms15, fms16, fms17, fms19, fms21, fnm, gls20, gls33, glsB, glsB1, hyl, orf1481, ptsD, sagA, scm, sgrA*	Rep17, RepUS15, Rep11a, Rep14a
TRCIO_35	*van*HAX	*aac(6')-Ii, ant(6)-Ia, aph(3')-III, aac(6')-aph(2''), msr(C), erm(B), tet(M), tet(L), dfrG*	*IS16, acm, bepA, ccpA, empA, empB, empC, fms11, fms13, fms14, fms15, fms16, fms17, fms19, fms21, fnm, gls20, gls33, glsB, glsB1, hyl, orf1481, ptsD, sagA, sgrA*	RepUS43, RepUS12, RepUS15 Rep17, Rep11a, Rep18b
TRCIO_36	*van*HAX	*aac(6')-Ii, ant(6)-Ia, aph(3')-III, aac(6')-aph(2''), msr(C), erm(B), erm(T), tet(M), tet(L)*	*IS16, acm, bepA, ccpA, empA, empB, empC, fms11, fms13, fms14, fms15, fms16, fms17, fms19, fms21, fnm, gls20, gls33, glsB, glsB1, hyl, orf1481, ptsD, sagA, scm, sgrA*	RepUS43, RepUS12, RepUS15 Rep17, Rep11a, Rep14a
TRCIO_40	*van*HAX	*aac(6')-Ii, ant(6)-Ia, aph(3')-III, aac(6')-aph(2''), msr(C), erm(B), tet(M), tet(L), dfrG*	*IS16, acm, bepA, ccpA, empA, empB, empC, fms11, fms13, fms14, fms15, fms16, fms17, fms19, fms21, fnm, gls20, gls33, glsB, glsB1, hyl, orf1481, ptsD, sagA, scm, sgrA*	RepUS43, RepUS12, RepUS15 Rep17, Rep11a

TRCIO_41	*van*HAX	*aac(6')-Ii, ant(6)-Ia, aph(3')-III, aac(6')-aph(2''), msr(C), erm(B), tet(M)*	*IS16, acm, bepA, ccpA, ecbA, empA, empB, empC, fms11, fms13, fms14, fms15, fms16, fms17, fms19, fms21, fnm, gls20, gls33, glsB, glsB1, hyl, orf1481, ptsD, sagA, scm, sgrA*	Rep11a, Rep17, RepUS15
TRCIO_42	*van*HAX	*aac(6')-Ii, ant(6)-Ia, aph(3')-III, aac(6')-aph(2''), msr(C), erm(B), tet(M), tet(L)*	*IS16, acm, bepA, ccpA, empA, empB, empC, fms11, fms13, fms15, fms16, fms17, fms19, fms21, fnm, gls20, gls33, glsB, glsB1, hyl, orf1481, ptsD, sagA, scm, sgrA*	RepUS43, RepUS12, RepUS15 Rep17, Rep11a, Rep2
TRCIO_43	*van*HAX	*aac(6')-Ii, ant(6)-Ia, aph(3')-III, aac(6')-aph(2''), msr(C), erm(B), dfrG*	*IS16, acm, bepA, ccpA, ecbA, empA, empB, empC, fms11, fms13, fms14, fms15, fms16, fms17, fms19, fms21, fnm, gls20, gls33, glsB, glsB1, hyl, orf1481, ptsD, sagA, sgrA*	RepUS15, Rep17
TRCIO_45	*van*HAX	*aac(6')-Ii, ant(6)-Ia, aph(3')-III, aac(6')-aph(2''), msr(C), erm(B), tet(M), tet(L), dfrG*	*IS16, acm, bepA, ccpA, empA, empB, empC, fms11, fms13, fms14, fms15, fms16, fms17, fms19, fms21, fnm, gls20, gls33, glsB, glsB1, hyl, orf1481, ptsD, sagA, scm, sgrA*	RepUS43, RepUS12, RepUS15 Rep17, Rep11a
TRCIO_46	*van*HAX	*aac(6')-Ii, ant(6)-Ia, aph(3')-III, aac(6')-aph(2''), msr(C), erm(B), tet(M), tet(L), dfrG*	*IS16, acm, bepA, ccpA, empA, empB, empC, fms11, fms13, fms14, fms15, fms16, fms17, fms19, fms21, fnm, gls20, gls33, glsB, glsB1, hyl, orf1481, ptsD, sagA, scm, sgrA*	RepUS43, RepUS12, RepUS15, Rep17, Rep11a
TRCIO_47	*van*HAX	*aac(6')-Ii, ant(6)-Ia, aph(3')-III, aac(6')-aph(2''), msr(C), erm(B), tet(M), tet(L), dfrG*	*IS16, acm, bepA, ccpA, empA, empB, empC, fms11, fms13, fms14, fms15, fms16, fms17, fms19, fms21, fnm, gls20, gls33, glsB, glsB1, hyl, orf1481, ptsD, sagA, sgrA*	RepUS43, RepUS12, RepUS15 Rep17, Rep11a
