# Supplementary material for: Surveillance and Characterization of Vancomycin-Resistant and Vancomycin-Variable Enterococci in a Hospital Setting
Source: Antibiotics (Basel). 2025 Aug 4;14(8):795. doi: 10.3390/antibiotics14080795 (PMC12383138; doi:10.3390/antibiotics14080795)
Supplement: Supplementary file 1 [file antibiotics-14-00795-s001.zip › Supplementary Files/Table S3-antibiotics-3720173.pdf]

Table S3. Molecular characterization of the Vancomycin-Variable enterococci (VVE) isolates.

ID VVE Isolates	Glycopeptide Resistance	Resistance genes	Virulence determinants	Plasmids
TRCIO_03	*van*HAX	*aac(6')-Ii, ant(6)-Ia, aph(3')-III, aac(6')-aph(2''), msr(C), erm(B), tet(M), tet(L), dfrG*	IS16, *acm, bepA, ccpA, empA, empB, empC, fms11, fms13, fms14, fms15, fms16, fms17, fms19, fms21, fnm, gls20, gls33, glsB, glsB1, hyl, orf1481, ptsD, sagA, sgrA*	RepUS43, RepUS12, RepUS15, Rep17. Rep11a
TRCIO_07	*van*HAX	*aac(6')-Ii, ant(6)-Ia, aph(3')-III, aac(6')-aph(2''), msr(C), erm(B), tet(M), tet(L), dfrG*	IS16, *acm, bepA, ccpA, empA, empB, empC, fms11, fms13, fms14, fms15, fms16, fms17, fms19, fms21, fnm, gls20, gls33, glsB, glsB1, hyl, orf1481, ptsD, sagA, sgrA*	RepUS43, RepUS12, RepUS15, Rep17, Rep11a
TRCIO_16	*van*A, *van*X	*aac(6')-Ii, ant(6)-Ia, aph(3')-III, aac(6')-aph(2''), msr(C), erm(B), dfrG*	IS16, *acm, bepA, ccpA, ecbA, empA, empB, empC, fms11, fms13, fms14, fms15, fms16, fms17, fms19, fms21, fnm, gls20, gls33, glsB, glsB1, hyl, orf1481, ptsD, sagA, scm, sgrA*	RepUS15, Rep18a
TRCIO_17	*van*T	*aac(6')-Ii, ant(6)-Ia, aph(3')-III, aac(6')-aph(2''), lsa(A), tet(M)*	-	RepUS43, Rep9b
TRCIO_29	*van*HAX	*aac(6')-Ii, ant(6)-Ia, aph(3')-III, aac(6')-aph(2''), dfrG, erm(B), tet(M), tet(L)*	*IS16, acm, bepA, ccpA, empA, empB, empC, fms11, fms13, fms14, fms15, fms16, fms17, fms19, fms21, fnm, gls20, gls33, glsB, glsB1, hyl, orf1481, ptsD, sagA, scm, sgrA*	RepUS43, RepUS12, RepUS15, Rep17 Rep11a, Rep18a
TRCIO_30	*van*Y	*aac(6')-Ii, ant(6)-Ia, aph(3')-III, aac(6')-aph(2''), tet(M)*	*acm, bepA, ccpA, empB, empC, fms15, gls20, gls33, glsB, glsB1, sagA*	Rep2, RepUS15
TRCIO_32	*van*HAX	*aac(6')-Ii, ant(6)-Ia, aph(3')-III, aac(6')-aph(2''), msr(C), erm(B), tet(M), tet(L)*	IS16, *acm, bepA, ccpA, empA, empB, empC, fms11, fms13, fms14, fms15, fms16, fms17, fms19, fms21, fnm, gls20, gls33, glsB, glsB1, hyl, orf1481, ptsD, sagA, sgrA*	RepUS43, RepUS12, RepUS15, Rep17, Rep11a, Rep14a
TRCIO_33	*van*T	*aac(6')-Ii, ant(6)-Ia, aph(3')-III, aac(6')-aph(2''), lsa(A), tet(M)*	*fms15*	Rep2, RepUS11, Rep6, Rep9a, Rep9b, RepUS43
TRCIO_37	*van*Y	*aac(6')-Ii, ant(6)-Ia, aph(3')-III, aac(6')-aph(2''), msr(C), erm(B), lsa(E), lnu(B), poxtA, tet(M), tet(L)*	*bepA ccpA, empB, fms11, fms13, fms14, fms15, fms16, fms19, fms21, gls20, gls33, glsB, glsB1, scm*	Rep2, RepUS1, RepUS15
TRCIO_38	*van*HAX	*aac(6')-Ii, ant(6)-Ia, aph(3')-III, aac(6')-*	IS16, *acm, bepA, ccpA, empA, empB, empC, fms11, fms13, fms14, fms15, fms16, fms17, fms19,*	RepUS43, RepUS12, RepUS15, Rep17,

		aph(2''), msr(C), erm(B), tet(M), tet(L), dfrG	*fms21, fnm, gls20, gls33, glsB, glsB1, hyl, orf1481, ptsD, sagA, sgrA*	Rep11a
TRCIO_39	*van*T	*aac(6')-Ii, ant(6)-Ia, aph(3')-III, aac(6')-aph(2''), erm(B), lsa(A), tet(M)*	-	RepUS43, Rep9b
TRCIO_44	*van*HAX	*aac(6')-Ii, ant(6)-Ia, aph(3')-III, aac(6')-aph(2''), msr(C), erm(B), tet(M), tet(L)*	IS16, *acm, bepA, ccpA, empA, empB, empC, fms11, fms13, fms14, fms15, fms16, fms17, fms19, fms21, fnm, gls20, gls33, glsB, glsB1, hyl, orf1481, ptsD, sagA, sgrA*	RepUS43, RepUS12, RepUS15, Rep17, Rep11a, Rep14a
