# Supplementary material for: Surveillance and Characterization of Vancomycin-Resistant and Vancomycin-Variable Enterococci in a Hospital Setting
Source: Antibiotics (Basel). 2025 Aug 4;14(8):795. doi: 10.3390/antibiotics14080795 (PMC12383138; doi:10.3390/antibiotics14080795)
Supplement: Supplementary file 1 [file antibiotics-14-00795-s001.zip › Supplementary Files/Table S6-antibiotics-3720173.pdf]

Table S6. Characterization of the different CTs and STs among the hospital wards.

ID Isolate	W (1-5)[1]	Species[2]	ST[3]	CT[4]
TRCIO_01	W-5	*E. faecium*	80	1
TRCIO_02	W-1	*E. faecium*	80	1
TRCIO_03	W-2	*E. faecium*	80	1
TRCIO_04	W-1	*E. faecium*	80	1
TRCIO_05	W-1	*E. faecium*	80	2
TRCIO_06	ICU	*E. faecium*	80	6
TRCIO_07	W-4	*E. faecium*	80	1
TRCIO_08	W-2	*E. faecium*	80	1
TRCIO_09	W-3	*E. faecium*	80	-
TRCIO_10	W-1	*E. faecium*	80	2
TRCIO_11	W-2	*E. faecium*	80	1
TRCIO_12	W-2	*E. faecium*	117	-
TRCIO_13	W-5	*E. faecium*	80	1
TRCIO_14	W-5	*E. faecium*	80	1
TRCIO_15	W-2	*E. faecium*	-	-
TRCIO_16	W-4	*E. faecium*	117	-
TRCIO_17	W-5	*E. faecalis*	-	4
TRCIO_18	W-1	*E. faecium*	80	2
TRCIO_19	W-3	*E. faecium*	80	6
TRCIO_20	W-2	*E. faecium*	80	1
TRCIO_21	W-1	*E. faecium*	80	1
TRCIO_22	W-4	*E. faecium*	80	-
TRCIO_23	ICU	*E. faecium*	1478	7
TRCIO_24	W-1	*E. faecium*	1478	5
TRCIO_25	W-2	*E. faecium*	1478	7
TRCIO_26	W-1	*E. faecium*	1478	5
TRCIO_27	W-2	*E. faecium*	80	1
TRCIO_28	W-4	*E. faecium*	80	1
TRCIO_29	W-5	*E. faecium*	80	1
TRCIO_30	W-4	*E. faecium*	1126	-
TRCIO_31	W-4	*E. faecium*	80	3
TRCIO_32	W-3	*E. faecium*	80	6
TRCIO_33	W-3	*E. faecalis*	-	4
TRCIO_34	W-2	*E. faecium*	80	-
TRCIO_35	W-4	*E. faecium*	80	1
TRCIO_36	W-3	*E. faecium*	80	-
TRCIO_37	W-4	*E. faecium*	971	-
TRCIO_38	W-1	*E. faecium*	80	1
TRCIO_39	W-1	*E. faecalis*	-	4
TRCIO_40	W-3	*E. faecium*	80	1
TRCIO_41	W-1	*E. faecium*	80	3
TRCIO_42	W-4	*E. faecium*	80	6

TRCIO_43	ICU	*E. faecium*	1478	-
TRCIO_44	W-3	*E. faecium*	80	6
TRCIO_45	W-1	*E. faecium*	80	1
TRCIO_46	W-3	*E. faecium*	80	1
TRCIO_47	W-5	*E. faecium*	80	1

[1]W (1-5): W-1: Hepatology Unit, W-2: Viral Immunodeficiency Unit, W-3: Immune systemic infections Unit, W-4: Respiratory system infectious diseases Unit, W-5: High Intensity Care Infectious Disease Unit; ICU: Intensive Care Unit.

[2]*E. faecium*: *Enterococcus faecium*; *E. faecalis*: *Enterococcus faecalis.*

[3]ST: Sequence Type.

[4]CT: Cluster Type.

[1]W (1-4): W-1: Hepatology Unit, W-2: Viral Immunodeficiency Unit, W-3: Immunodepressed Sistemic Infection Unit, W-4: UOC MIAR, W-5: High-intensity and Care Unit, ICU: Intensive Care Unit;

[2]Species: *E. faecium*: *Enterococcus faecium*, *E. faecalis*: *Enterococcus faecalis*;

[3]ST: Sequence Type;

[4]CT: Cluster Type.

CT-1 was the most prevalent, making up 42.5% of the samples and was found in all wards except the ICU. CT-1 was mostly prevalent across all hospital wards, containing 25% (5/20) of isolates found in W1, and 25% (5/20) in W2. In contrast, CT-2 is the most prevalent in W-1, containing 100% of the samples. The isolates from the ICU cluster only in CT-6 and CT-7 (50%, 20%).
