# Supplementary figures and images for: Surveillance and Characterization of Vancomycin-Resistant and Vancomycin-Variable Enterococci in a Hospital Setting
Source: Antibiotics (Basel). 2025 Aug 4;14(8):795. doi: 10.3390/antibiotics14080795 (PMC12383138; doi:10.3390/antibiotics14080795)

**Figure S1.** Heatmap showing the Average Nucleotide Identity (ANI) among the strains.

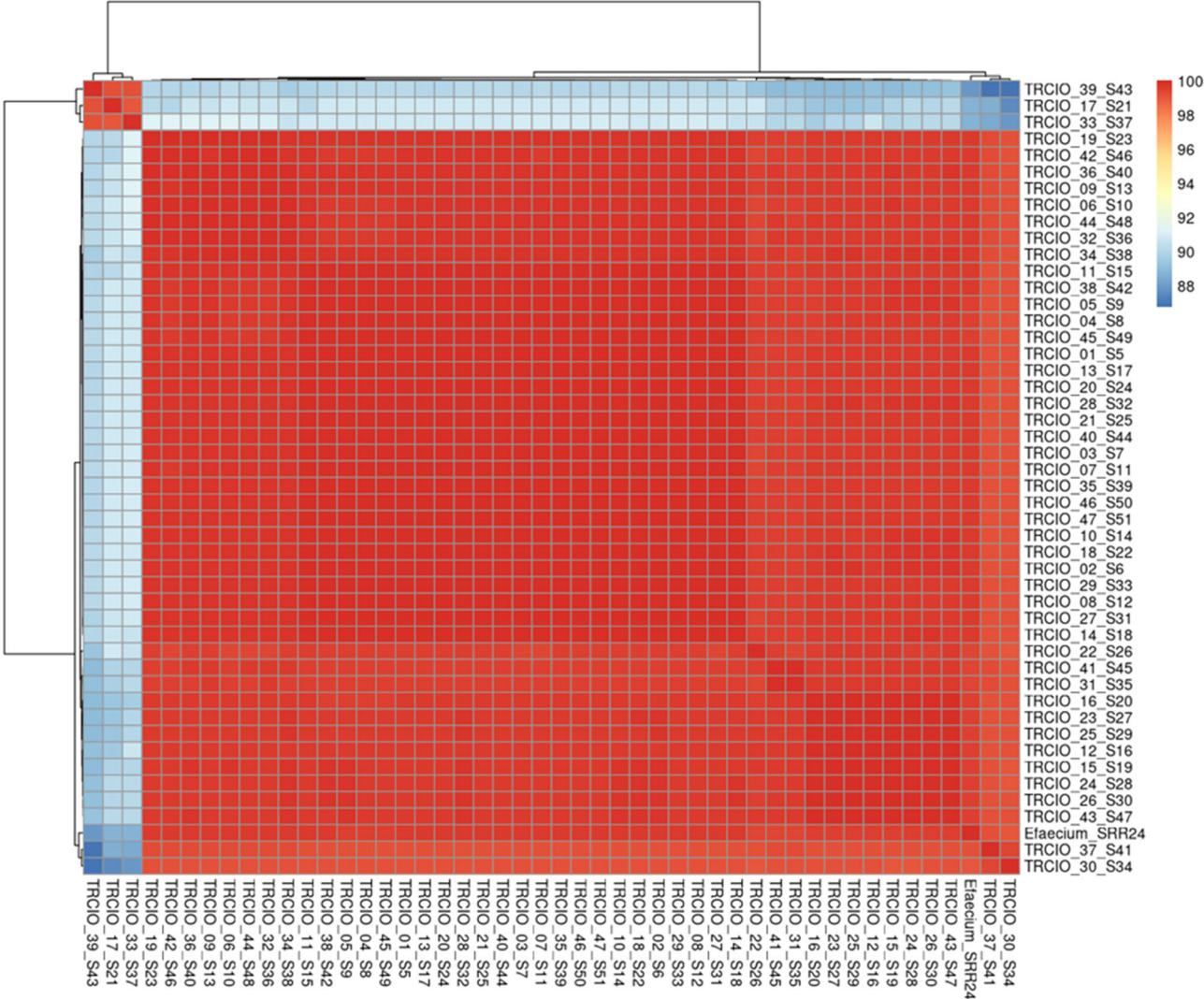

Supplement: Supplementary file 1 [file antibiotics-14-00795-s001.zip › Supplementary Files/Figure S1-antibiotics-3720173.pdf]
